# Therapeutic Communication Experiences of Nurses Caring for Patients with Hematology

**DOI:** 10.3390/healthcare10122403

**Published:** 2022-11-30

**Authors:** Hyun-Jung Lee, Bom-Mi Park, Mi-Jin Shin, Do-Yeon Kim

**Affiliations:** 1 Department of Nursing, The Catholic University of Korea, Seoul ST. Mary’s Hospital, Seoul 06591, Republic of Korea; 2 Department of Nursing, Konkuk University, Chungju-si 27478, Republic of Korea

**Keywords:** therapeutic communication, patients with hematology, nurse, phenomenology, focus group interview

## Abstract

Nurses who take care of patients with hematology have more difficulty in therapeutic communication. The aim of this study is to explore the therapeutic communication experiences of nurses caring for patients with hematology and the meaning of the essential structure of therapeutic communication. Colaizzi’s phenomenological method was applied to explore the essential structures and meanings of therapeutic communication in depth through a focus group interview. The interview was conducted at a tertiary care hospital with 20 nurses caring for patients with hematology. As a result of the analyses, 22 themes, 14 theme clusters, and 5 categories were derived. The categories derived from the analyses included “acquiring core competencies as nursing professionals”, “improving patient-centered nursing performance”, “forming a partnership treatment relationship”, “obtaining clinical performance skills to solve problems”, and “preparing efficient system improvement”. Based on this study’s results, it will be possible to provide high-quality nursing to patients by improving the therapeutic communication ability of nurses caring for patients with hematology. In addition, it will be the basis for the development of a nurses’ therapeutic communication promotion program for nurses caring for such patients.

## 1. Introduction

Therapeutic communication is defined as an interaction aimed at improving the emotional and physical problems of patients [1,2]. Therapeutic communication includes open-ended questions, listening, reflection, silence, clarity, nonverbal or verbal signals, identifying and providing evidence, and summarizing the tone of emotion [3]. For nurses, in particular, communication can be considered an essential component of nursing expertise that can enable them to understand, evaluate, and focus on each patient [4,5]. A nurse’s effective therapeutic communication represents their ability to know what to say and do during interactions with a patient [6]. The patients’ satisfaction with hospital life varies according to the nurses’ level of communication [7]. Communication increases patients’ knowledge, enabling them to use self-health techniques that ultimately affect their health and well-being [8]. Ultimately, therapeutic communication between nurses and patients can foster positive treatment results through certain interactions [4].

Patients come to the hospital due to illness and suffer mentally and physically due to changes in the unfamiliar environment of the hospital [9]. Patients with hematology must accept the consequences of a life-threatening disease after the initial diagnosis as well as endure the side effects of various tests and treatments, along with changes in their living conditions and lifestyles [10]. Such patients, in particular, initially exhibit poor communication, such as avoiding talking about their disease [10]. However, the rate of progression of hematological diseases is fast, often having fatal consequences; therefore, after receiving treatment, patients may continue to rely on nurses and continuously ask questions or have concerns about the uncertain number of treatments [11]. This is because patients feel comfortable when nurses approach and talk to them, and they hope that the nurse will spend considerable time communicating with them [10]. However, in a clinical setting, patients may perceive a lack of therapeutic communication with the nurses due to the insufficient number of nurses, overwork of nurses, lack of knowledge of therapeutic communication with nurses, and the patient’s own anxiety and suffering [2].

Notably, one study reported that the lack of therapeutic communication by nurses was associated with a lack of repeated acquisition of therapeutic communication skills [4]. In addition, nurses providing care to patients with hematology have difficulty communicating with patients during crisis situations, such as at the first diagnosis or if the disease recurs [12]. Moreover, during the coronavirus disease 2019 (COVID-19) pandemic, a study reported that patients perceived more barriers to communication due to the wearing of face masks [13]. Communication between patient and nurse in clinical situations occurs through interaction and may vary depending on the situation and personal characteristics [14]. Therefore, nurses should be able to communicate with patients and understand their needs and emotions [15].

Most of the advanced qualitative studies on nurses’ therapeutic communication to date have focused on nurses caring for cancer patients, nursing students, and therapeutic communication by hematology oncologists; however, qualitative studies on nurses caring for hospitalized patients with hematological cancer at tertiary hospitals are scarce. Therefore, in this study, we aim to explore the essential structures and meanings of the therapeutic communication experiences of the nurses caring for patients with hematology. To elucidate the comprehensive experiences of nurses caring for patients with hematology, focus group [16] interviews, aimed at extensively exploring individual experiences, sharing ideas, and gathering opinions on common problems, were conducted, confirming nurses’ difficulty in providing therapeutic communication.

## 2. Materials and Methods

### 2.1. Design

In this study, we employed focus group interviews to explore the therapeutic communication experiences of nurses experienced in caring for patients with hematological cancer. A qualitative design was adopted by applying the phenomenological method of Colaizzi [17], a rigorous and qualitative approach to finding, understanding, explaining, describing, and revealing new topics and intertwined relationships [17], to examine the structure and meaning of therapeutic communication.

### 2.2. Selection of Participants

Participants in this study were 20 nurses working at a tertiary care hospital in a metropolitan city in Korea who had experience caring for patients with hematological cancer. They felt keenly about communication problems with blood cancer patients and wanted to actively participate. We selected 10 nurses with more than five years of clinical experience and 10 with less than five years of clinical experience through an announcement by the Ministry of Nursing; those who fully understood this study and agreed to participate were sampled. The participants’ general characteristics included age, gender, education, clinical experience with patients of a hematology cancer ward, and total clinical experience. The nurses in the ward were usually young (the median age was 29 years), and all 20 nurses were women. The nurses’ educational background was a bachelor’s degree or higher, and the average length of clinical experience in the hematology cancer ward was four years and eight months. Their average length of clinical experience was five years and eight months (Table 1). 

### 2.3. Procedure

Data collection for this study was conducted after obtaining approval from the Institutional Review Board. The researcher submitted a letter of cooperation to the tertiary care hospital’s nursing department. Thereafter, the researcher conducted non-face-to-face interviews in accordance with COVID-19 response guidelines. Additionally, before obtaining the consent form, he met each participant one-on-one and explained the research. 

The interview was conducted at the time promised in a place where the interview could be kept confidential, such as a quiet place for non-face-to-face interviews. Based on the main questions of the interview guide, which were written in advance, additional questions were asked if necessary so that the research participants could fully talk about their experiences. Participant cards with information on the participants were created and filled out before the interview, which lasted approximately an hour. When the interview began, the participants were notified of the start and were thereafter recorded. Parts of the interview that were difficult to understand were clarified by the researcher asking additional questions at the end of the session. When it was determined that the group interview did not yield any new content and had reached saturation, the data collection was terminated. 

The interview questions were in five formats: starting, introduction, transition, main, and closing questions [18]. Additionally, the questions were verified by two nurses in their 10th year of experience as to the validity of the contents of the interview. First, at the start of the interview, the interviewer softened the atmosphere through everyday conversation. Using a semi-structured questionnaire, the interview was started with the following introductory open question: “What comes to your mind when you think of therapeutic communication?”. The transition question was, “What has been your experience in therapeutic communication with patients?”. The main questions were, “What difficulties did you experience in therapeutic communication with patients in clinical practice?”, “What qualities do you think nurses should possess for therapeutic communication?”, and “What effects do nurses have on clinical communication?”. These were followed by, “What education do you think is necessary for nurses in relation to therapeutic communication promotion?”, and the closing question, “We discussed therapeutic communication promotion in clinical practice; is there anything else you want to say?”.

The interview was conducted by asking a series of questions based on the participants’ answers. The data were transcribed immediately after the interview by the co-researcher in the participants’ language, and the responsible researcher checked the recorded data and confirmed the final transcript. All personal information of the participants was anonymized by numbering.

### 2.4. Data Analysis

The collected data were analyzed according to the phenomenological method of Colaizzi [17]. First, after repeatedly reading the transcribed data, the researcher identified the participants’ experiences as well as their overall meanings, contents, and tones. Second, meaningful statements (words, phrases, and sentences) were extracted from the transcribed interview contents. Third, the researchers exchanged opinions on and extracted the common elements of the derived statements. Fourth, the meaning of the derived statements was re-stated in the researcher’s language. Fifth, themes encompassing the entire experience were selected from the constructed meaning. Sixth, the meaning was reaffirmed by reviewing the data, and based on the derived subjects, it was categorized into theme clusters. Finally, after classifying the derived meanings and repeatedly sharing opinions, the structure and collection of the final categories were created. After verifying reliability and validity, the essential structure of the research phenomenon was described collectively.

### 2.5. Preparation of Researchers and Feasibility of Research

The responsible researcher has completed the development of nursing theory and qualitative research methodology in a doctoral course to lay the foundation for the theory and practice of qualitative research. Additionally, to expand the understanding of phenomenological research methods, efforts were made to elucidate the nature of the phenomenon by reading books related to phenomenology and qualitative research papers. Furthermore, all researchers tried to exclude their preconceptions as much as possible in order to have a neutral attitude during the study.

To secure the validity of the study, we followed the truth value, applicability, consistency, and neutrality criteria by Lincoln and Guba [19]. To secure the truth value, the recorded data were transcribed by the researcher and research assistant while listening to the recorded file. The recorded data were compared with the transcribed data while listening repeatedly two or more times. To secure applicability, two clinical nurses who were not participants in the study but had nursing experience with patients in similar situations were consulted. We intended to secure the consistency of data analysis by receiving feedback on the concepts and categories from two nursing professors with qualitative research experience. To secure neutrality, the research was conducted by excluding prejudice as much as possible. 

### 2.6. Ethical Considerations

This study was conducted after obtaining approval from the Institutional Ethics and Life Committee of the Catholic University of Seoul Medical Center (approval number: KC21EASI0657). To guarantee the autonomy of the participants, the purpose and research method of the study were explained to them before the interview; additionally, written consent was obtained after explaining that they could withdraw their participation at any time. To ensure anonymity in the analysis process, the data were stored under numbers instead of participants’ names.

## 3. Results

As a result of analyzing the interview data of the study participants, 22 themes, 14 theme clusters, and 5 category collections with more comprehensive meanings were extracted. The categories that were derived and analyzed are: “acquiring core competencies as nursing professionals”, “improving patient-centered nursing performance”, “forming a partnership treatment relationship”, “obtaining clinical performance skills to solve problems”, and “preparing efficient system improvement” (Table 2).

### 3.1. Category 1: Acquiring Core Competencies as Nursing Professionals

Participants stated that to communicate therapeutically with patients, they require certain core competencies as nursing professionals. They said that nurses should first approach the patient with an empathic and proactive attitude and that job satisfaction and self-efficacy increase through their practical work. Furthermore, they considered it important to have an attitude of not getting swayed by emotions during an emergency.

#### 3.1.1. Empathize and Take a Proactive Attitude from the Patient’s Perspective

The participants of this study agreed that therapeutic communication should be understood from the patient’s perspective based on their values. Additionally, they considered that the patient’s treatment effect would improve if they had sufficient time to initially approach and sympathize with the patient as medical staff.

“I think communication involves knowing my values first, to empathize from the patient’s point of view, and to understand the patient in an open manner”

(Participant 11)

“Many people suffer from mental illness as well as physical illness, and the more time we spend in sympathizing, asking, and listening first, the more effective it will be to treat patients”

(Participant 4)

In this study, nurses said that they should have a proactive response or resolution to the patient’s complaints. To communicate therapeutically, participants stated they should first be able to quickly recognize the symptoms that the patient complains about. Further, nurses should improve their treatment ability to solve nursing problems, which should be followed by therapeutic communication. One participant reported that a patient expressed gratitude for a quick response.

“When I look at the patient’s needs, I think I need an attitude of active participation”

(Participant 17)

“I think I can quickly recognize the symptoms that the patient complains of, solve the nursing problem, and then have smooth therapeutic communication if empathy and listening skills are added”

(Participant 8)

“While going through the morning rounds, we get caught up on the parts that we couldn’t see and lacked, and when immediate treatment is provided, they trust us more and say ‘thank you’ later”

(Participant 3)

#### 3.1.2. Improve Satisfaction and Self-Efficacy through Work

The participants of this study said that it felt rewarding to communicate with patients in a comfortable atmosphere. Additionally, through therapeutic communication with patients, the nurses’ work satisfaction increased when patients opened up.

“Through therapeutic communication, nurses gain a lot of confidence in their nursing behavior, and their satisfaction with the nursing job increases, improving their efficiency in nursing”

(Participant 9)

“When I communicate therapeutically, I feel proud as a nurse and my job satisfaction increases when the patient opens up”

(Participant 7)

“I also feel that my satisfaction increases when I communicate with patients, and I perform my work with a more proactive attitude”

(Participant 6)

The nurses stated that their self-efficacy was formed by helping patients and that their self-efficacy increased through therapeutic communication as the patients appeared to be emotionally stable.

“I think my self-efficacy increases a lot because I can also help patients with their emotions”

(Participant 18)

“I think healing communication increases my self-efficacy and confidence in my work makes me better at therapeutic communication with patients. By doing this, it was good when the patient looked a little stable and expressed gratitude when I empathized with their feelings”

(Participant 17)

#### 3.1.3. Staying Centered So That Nurses Do Not Get Carried Away

In this study, the participants indicated that it is important to separate emotions from patients and maintain them consistently without overindulgence in emotional situations. Furthermore, they did not react emotionally when a patient’s complaint came at a busy time.

“There are a lot of patients dying. Therefore, I think it is necessary to control your mind to keep your emotions consistent without becoming overly immersed”

(Participant 18)

“I was having a difficult time adjusting [to] a room with the staff and coordinator of the hospital room because of a problem, and one patient was severely complaining. At that time, I treated the patient calmly rather than with anger. Nurses get hurt a lot, too. I’m dealing with these patients. Experiences that are not influenced by emotions are important”

(Participant 2)

### 3.2. Category 2: Improving Patient-Centered Nursing Performance

Participants said that patient-centered nursing performance should be improved to communicate therapeutically with patients. Moreover, it is important to actively motivate patients to participate in their treatment, relieve their anxiety through rapport formation, and improve the trust in nurses.

#### 3.2.1. Promote Active Patient Engagement

In this study, the participants reported that positive effects could be realized through patient-centered treatment. At first, patients might have tried to reject it, but if the nurses actively participated with the patient, they could later see the patients participating in their own treatments.

“If patients actively participate in self-treatment and brush their teeth, gargle, and perform hand hygiene on their own, it will have a positive effect on preventing infection and recovering mucosal inflammation”

(Participant 2)

“In the case of bedridden patients, although they lie still and do nothing, they actively participate in the treatment. At first, [he] couldn’t brush [his] teeth, but when I told him and wiped his face, the patient said he would wash his face and wash himself later. This support from medical personnel seems to provide patients with a sense of self-efficacy”

(Participant 3)

#### 3.2.2. Relieving Anxiety through Rapport Formation

In this study, the participants reported that it was possible to relieve patient anxiety by encouraging an active expression of nursing needs. Patients express their anxiety while forming a rapport with the nurse, and they are able to resolve their anxiety based on the nurse’s listening and empathy.

“If you express exactly what the patient wants through rapport formation, it seems to be more effective in treatment of and relieving the patient’s anxiety”

(Participant 5)

“I believe patients’ anxiety is reduced through therapeutic communication”

(Participant 5)

“When a patient expresses their feelings and condition to a nurse, the anxiety is relieved just by expressing it, and the nurse’s empathy and listening can form a rapport, bond, and give credibility”

(Participant 10)

In this study, the participants said that patient anxiety could be reduced by creating an empathetic atmosphere in a tense situation. They further reported that there was another way to relieve patients’ anxiety with the help of other departments.

“A patient couldn’t sleep at all on the day of the transplant because of anxiety. When I asked him if he slept well in the morning, he said he didn’t sleep at all. So, I sympathized with the patient’s situation by saying, ‘Usually, the experience of treatment can come to mind and [patients] think a lot the day before the transplant’”

(Participant 15)

“He was very depressed and anxious. He had some mental health problem, but he was a patient who refused to take medication. Nurses, of course, continued to listen to the patient with empathy, but through several interviews with the nun at the hospital, the patient’s anxiety was reduced and mind was opened, so they could take prescribed drugs well”

(Participant 11)

#### 3.2.3. Increased Confidence in Nursing Work

This study’s participants indicated that trust in nurses could be improved by providing professional practical skills and knowledge. The participants said that they had the expertise and an open attitude toward communicating therapeutically with patients to improve trust.

“I think providing nursing through practical skills and knowledge can improve patients’ trust”

(Participant 8)

“Above all, nurses need to have expertise in building trust. Basically, if you accept and communicate with the patient in an open manner with professional nursing knowledge, a trust relationship seems to form naturally”

(Participant 11)

“When patients trust nurses through therapeutic communication, they seem to be better able to fulfill what they have to do on their own”

(Participant 8)

### 3.3. Category 3: Forming a Partnership Treatment Relationship

In this study, the nurses said that a partnership treatment relationship should be formed to communicate therapeutically with patients. The participants said that they could sympathize and listen to the patients at their level and form a partnership treatment relationship by respecting patients as human beings and the interaction between patients and nurses.

#### 3.3.1. Empathize and Listen to the Patient at Their Level

In this study, the participants considered it necessary to sympathize and listen to individual patient cases. They said it was important to form a consensus through an attitude of caring for the family. Additionally, nursing from the patient’s point of view should be considered.

“I think empathy is what nurses need the most. Most of the time, I care for older people, so if you’re my mother… If it’s my dad… ‘I’m going to nurse him like this’”

(Participant 4)

“Nurses are busy at work, but I think nursing should be done based on the attitude of empathizing and listening to patients”

(Participant 6)

Further, participants reported that it was necessary to provide stability when a patient stays in the hospital for the first time. The nurses said that they provided therapeutic communication by providing necessary information to patients staying in the hospital for the first time based on their clinical experience.

“In some cases, children are diagnosed with leukemia because they think it’s just a cold, and in others, their guardians usually blame themselves. In that case, they say, ‘It’s not the guardian’s fault,’ and they can treat it. It was good when I said, ‘If you show the patient how you overcome it together, and not the mother having a hard time’”

(Participant 13)

“When I provide information based on my clinical experience to patients who come to the hospital and have been diagnosed for the first time, I feel like I am communicating therapeutically”

(Participant 9)

#### 3.3.2. Respect for the Patient as a Human Being

The participants said that it is necessary to provide nursing care by respecting patients as humans and that through therapeutic communication with patients, they understand and sympathize more with the patient as a person.

“I think we need to look at the patient as a person, ask how he feels today, and whether he is in a better condition than yesterday, and ask them questions that allow them to be seen as a person and not a patient”

(Participant 10)

“It felt like I was understanding, empathizing, and communicating with patients in different situations in a therapeutic way. I think my personal side is also developing”

(Participant 19)

#### 3.3.3. Patient–Nurse Interaction

In this study, we found that a positive synergy effect occurred due to the interaction between patients and nurses. Participants said that it was particularly effective in a busy nursing environment.

“When I communicate therapeutically with patients, I feel like I’m gaining strength and healing. It’s therapeutic communication for patients, and I think sometimes I try to communicate to gain strength, and I tend to take care of patients. So, in the end, when the patient and the nurse really communicate, I think they’re treating each other” (Participant 1). “I think it’s important that we look at the patient’s condition first and ask questions while we’re rounding to make him feel that he’s getting attention. The patient also seems to have an effect on the treatment effect by telling them about their uncomfortable situations first”

(Participant 13)

In this study, the nurses reported that patients were affected by nurses’ behaviors.

“I think every nurse’s behavior affects the interaction relationship with the patient”

(Participant 3)

“I think it is also important to allow nurses and patients to work together to solve patient health problems”

(Participant 3)

### 3.4. Category 4: Obtain Clinical Performance Skills to Solve Problems

The participants reported that to achieve therapeutic communication skills, a clinical performance ability for problem-solving should be acquired. For this purpose, nurses should develop themselves and provide an opportunity for self-development for problem-solving and scenarios on how to cope in a similar situation. 

#### 3.4.1. Self-Development

The participants stated that self-development should be achieved through the acquisition of professional knowledge and skills. Moreover, accurate information delivery and continuous studying are necessary for therapeutic communication.

“As a nurse, I think that such professional knowledge and skills should be the basis, and then communication skills should be included”

(Participant 7)

“I had a difficult time when I was intimidated because I didn’t know the answer when a patient asked me a question. I think it is necessary to study the disease continuously. That way, I think I will be able to communicate therapeutically with the patient, gain confidence, and have an attitude of listening”

(Participant 12)

Nurses in this study said that the ability to determine and solve nursing needs based on priorities is necessary. Having the ability to resolve a patient’s symptoms is also a method of therapeutic communication.

“I think accurate care for the nursing problems that patients require should be given first. I think it is more effective to control pain first in patients who are sick and then to communicate therapeutically. I also think the nursing ability to identify and respond to the patient’s needs first is necessary”

(Participant 20)

“For therapeutic communication, I think we need the ability to solve the symptoms that the patient complains of first. There was a patient who had delusions of damage or delirium during the transplant and was restless because he couldn’t sleep. We listened first to the constant appeals and complaints, and then consulted with the doctor to add drugs and allow the caregiver to stay stable”

(Participant 3)

#### 3.4.2. Provide Indirect Experience Opportunities for Contextual Scenarios

The participants of this study said that they felt the need to receive educational opportunities depending on the situation and that they wanted to communicate effectively with patients by receiving education on how to communicate with them.

“I think we need an education program that teaches us how to listen and respond when talking to patients”

(Participant 13)

“I’ve taken communication education at a hospital, and I don’t think it’s often, but I hope there’s a regular one”

(Participant 15)

In the group interview, difficulty in communicating according to various situations was expressed. Participants reported that they felt awkward and worried when communicating with patients. Therefore, there were several demands for education on how to communicate based on certain situations.

“To be honest, it’s very hard for me to start talking. For example, if you are a guardian of new patients and you are crying, or if the guardian is having a hard time due to the death of the patient, it would be good to receive training on how to start conversation and what words are helpful to the other person”

(Participant 10)

“There’s a patient who doesn’t say a word. In such a situation, I am worried about how to approach and how to open my heart”

(Participant 4)

### 3.5. Category 5: Preparing Efficient System Improvement

The group interview revealed that the efficient hospital system should be improved to communicate therapeutically with patients. Participants said that they should streamline the operations of nursing personnel, share experiences to improve the quality of nursing, and cooperate with other departments.

#### 3.5.1. Efficient Operation of Nursing Staff

Participants reported that the number of patients a nurse is in charge of should be adjusted according to the patients’ symptom severity. They added that it was necessary to adjust the number of patients for full-time nursing, indicating that the number of assigned patients at work was large and there were cases where they wanted to communicate therapeutically with one patient but became frustrated because it did not go as intended.

“If the number of patients in charge per nurse is low, I think we can provide more full-time care to each patient”

(Participant 8)

“My work is so busy, and I’m overwhelmed with work that I can’t afford it myself. I want to communicate therapeutically with the patient, but sometimes I wonder if they are serious about it”

(Participant 18)

In this study, the participants expressed that nursing work is difficult with increased severity. Further, they stated that when they see patients with hematology, they are pressed for work and time and are thus unable to respond suitably; if they are supplemented with more manpower, they will have sufficient time to conduct better therapeutic communication.

“There are so many symptoms that patients with hematology complain of, and the severity is high; so, I think it is necessary to make efforts to reduce the number of patients per nurse”

(Participant 6)

“I think we can better communicate the therapeutic communication we want to only when we replenish the nursing workforce so that I can have time for the patient as well”

(Participant 5)

“On days when I come to work as an over-member, I think I can definitely listen to patients more and take care of even the smallest things for them, so I can communicate well in therapy”

(Participant 2)

#### 3.5.2. Share Experiences to Improve Nursing Quality

In this study, effective communication methods were shared according to the situation. Participants expressed their desire to communicate effectively with patients by sharing other people’s experiences.

“I think it’s very important to communicate about situations that a patient may experience such as discomfort and dying conditions. Even if it’s not a simulation, I think it’s better for more experienced people to share how to communicate in such a situation”

(Participant 5)

#### 3.5.3. Cooperation with Other Departments

In this study, the nurses said they tried to share information with other departments regarding patient treatment. They said that they would be able to identify enough patients by sharing the parts of the treatments that they did not know about while nursing and communicating therapeutically based on the patients’ conditions.

“I think it is necessary to promote communication with other medical staff. If we share the patient’s treatment plan that the doctors’ team is thinking about, and what the nurse does not know, such as what we discussed with the rehabilitation and social work teams, we will be able to know the patient’s condition well and nurse accordingly”

(Participant 10)

## 4. Discussion

This study was conducted to explore the therapeutic communication experiences of nurses caring for patients with hematology. As a result of an analysis using Colaizzi’s [17] phenomenological method, 5 categories and 14 theme clusters were derived from the data. The five categories were “acquiring core competencies as nursing professionals”, “improving patient-centered nursing performance”, “forming a partnership treatment relationship”, “obtaining clinical performance skills to solve problems”, and “preparing efficient system improvement”.

In the category of “acquiring core competencies as nursing professionals”, the themes were: “empathize and take a proactive attitude from the patient’s perspective”, “improve satisfaction and self-efficacy through work”, and “stay centered so that nurses do not get carried away”. This category revealed that nurses approach nursing from the patient’s perspective and improve their self-efficacy through work, indicating that emotions during nursing work should be separated from professional skills. The finding of nurses gaining understanding through the patients’ viewpoint based on their values was similar to a study that said therapeutic communication skills such as reflection, empathy, and listening were necessary for an effective therapeutic relationship [20]. The present participants expressed that their self-efficacy was formed by helping patients, and it was necessary to isolate their emotions during emotional situations. This is similar to a previous study that reported that nurse job satisfaction, work commitment, self-efficacy, self-regulation, and prediction technology development increase patient satisfaction [21]. Additionally, emotional distancing can result in providing the best care to patients while protecting the mental health of nurses, which helps to reduce nurses’ emotional labor and maintain professionalism [22]. 

However, there is insufficient institutional supplementation or education for nurses regarding separating emotions or inculcating empathy for patients in hospitals. Therefore, to acquire core competencies as a professional nurse, it is necessary to not only improve the nurse’s active response method to patients and the rewarding feeling when caring for patients but also to support nurses in not becoming overwhelmed by their emotions. 

In the category of “improving patient-centered nursing performance”, the derived themes were: “promote active patient engagement”, “relieving anxiety through rapport formation” and “increased confidence in nursing work”. In this category, the patient’s active participation in treatments showed a positive effect, and communication from the patient’s perspective resulted in reduced anxiety.

This indicates that confidence-building between patients and nurses allows patients to communicate freely [23]. Moreover, confidence-building serves as the basis for a therapeutic relationship and allows patients to respect and comply with the nurse’s judgment [20]. Furthermore, nurses reported that patients’ confidence in nurses improved through the utilization of practical skills and knowledge, and prior results show that patients can obtain necessary medical information through communication with nurses while improving treatment performance and health management [24]. 

In recent studies, patient participation in the medical system is a legal right, requiring patients to participate in decisions related to their healthcare planning, effectiveness, and evaluation [25]. It has been reported that the concept of patient-centeredness is important in providing integrated treatment [26]. Therefore, for therapeutic communication, patients should actively participate in treatment, and nurses should provide patients with opportunities to express their nursing needs. 

Additionally, nurses need to make individual efforts to improve standard practical skills and proficiency in work to improve professionalism. 

In the category of “forming a partnership treatment relationship”, the derived themes were: “empathize and listen to the patient at their level”, “respect for the patient as a human being”, and “patient–nurse interaction”. In this category, it was found that nursing care should be provided with respect to the patient as a person and with an attitude of caring to the family. In addition, even during busy nursing situations, the interactions between a patient and a nurse may have a positive effect, and the patient may be affected by the nurse’s behavior. This is in line with previous studies that have reported that therapeutic communication can be a small spark that generates strong energy and clarifies the aims pursued by the nurse and the patient [24]. The patient’s response may vary depending on the characteristics of the interaction, including the communication used by the nurse [27]. Therefore, for therapeutic communication, the nurse pays more attention to the patient and communicates directly to empathize with the patient’s pain [28]. However, research on the interaction between patients and nurses is insufficient [25,27]. Therefore, further research on the interaction between patients and nurses is required. 

In the category of “obtaining clinical performance skills to solve problems”, the derived themes were: “self-development” and “provide indirect experience opportunities for contextual scenarios”. In this category, the necessity of providing educational opportunities for nurses appeared to be due to self-development through the acquisition of professional knowledge and skills and experiencing difficulties in communication in various situations. It is important to understand how nurses continue to improve their professionalism as their knowledge expands and expectations for better outcomes increase in an era of increasing demand for accountability in healthcare [29]. Similar to previous studies [24], the development of educational programs and effectiveness verification studies is necessary to promote therapeutic communication. In today’s technical and complex medical environment, the educational level and years of experience of nurses are important not only for them to continue providing effective care but also for their expectations regarding lifelong learning [29].

In the category of “preparing efficient system improvement”, the derived themes were: “efficient operation of nursing staff”, “share experiences to improve nursing quality”, and “cooperation with other departments”. This category highlights the need to adjust the number of patients for the realization of full-time nursing and to adjust the number of nurses according to the patient’s increased symptom severity. In addition, it is important to provide nurses with opportunities to share effective communication methods based on individual situations, along with sharing information with other departments for patient treatment. This indicates that patients want nurses to spend more time helping them solve problems [30]. Due to lack of time, nurses may ask superficial questions during interviews, making patients feel the impatience of the nurses, especially in the case of new nurses who are pressed for time, with patients considering it useless to talk to nurses as they appear busy [24]. Nursing in oncology particularly requires innovative recruitment strategies, onboarding and continuous education programs, industrial safety measures, and anti-burn interventions as nurses are scarce, and there are recruitment barriers (e.g., awareness of demanding specialties with complex treatments and dangerous work environments) and burnout [31]. Therefore, it is critical to provide institutional support for nurses to self-develop through the acquisition of professional knowledge and skills and to provide indirect experience opportunities through the development of situation-specific scenarios. Additionally, it is necessary to systematically provide opportunities for communication by connecting experienced nurses with new nurses to educate them on effective communication according to different situations. Furthermore, interaction and teamwork between experts are important, and cultural conditions and contextual determinants should be included at this time [32]. Moreover, sufficient knowledge of each other’s work, a culture of mutual respect, the recognition of each other’s expertise and capabilities, and free and open information exchange are necessary, in addition to sufficient time and resources for establishing effective and continuous relationships between care providers [33,34]. 

As shown by previous studies, organizational system design is needed to foster better partnerships and information sharing and to support integration between providers, healthcare professionals, and patients [33]. Further, it will be necessary to improve the system for sharing information with other departments for the more efficient treatment of patients. Nurses are the best professionals to coordinate patients’ care because they spend more time with patients than any other healthcare professional [31]. In particular, hematology nurses should be able to form appropriate therapeutic communication with patients by sensitively examining the patient’s condition, which can change rapidly depending on the progression of the disease. However, nurses who take care of patients with hematology in a clinical setting see many patients and are, thus, in a hurry to perform nursing tasks rather than conduct therapeutic communication. Additionally, when caring for patients with hematology in clinical settings, although the nurses know that therapeutic communication is necessary, they may not know the appropriate method, hence experiencing difficulties and raising the demand for therapeutic communication education and training. Therefore, for nurses to effectively communicate therapeutically with patients with hematology, it is necessary to train them in various approaches while considering the patient’s symptoms and emotions [24]. 

The limitations of this study are as follows. First, it was difficult to confirm whether therapeutic communication was conducted in accordance with the stage and disease type of patients with hematology. Second, the findings of this study should be generalized with caution as the research results were obtained from a single institution. Third, the average experience of caring for patients with hematology was four years and eight months, and it was difficult to confirm whether there was a gap in therapeutic communication quality. 

This study is meaningful in that it is the first to examine the therapeutic communication experience of nurses caring for patients with hematological cancer. Additionally, this study does not represent a specific sample, and the data and results of this study can help new nurses in providing care to patients with hematological cancer.

## 5. Conclusions

In this study, a focus group interview was conducted and analyzed using a phenomenological method to gain an in-depth understanding of the therapeutic communication experiences of nurses caring for patients with hematological cancer. It was confirmed that the nurses possessed core competencies for conducting therapeutic communication as nursing professionals, formed partnerships at the patient center, acquired clinical performance capabilities, and implemented efficient institutional improvements to solve problems. This study’s findings can enable the provision of high-quality nursing to patients by improving the therapeutic communication ability of nurses caring for patients with hematological cancer. In addition, the findings can be used as basic data for the development of a therapeutic communication program through the interactions between patients and nurses. Additionally, based on the results of this study, follow-up research should develop a standardized tool that can measure therapeutic communication ability. Moreover, based on the theory, it is necessary to develop a therapeutic communication promotion program for nurses caring for patients with hematology cancer.

## Figures and Tables

**Table 1:** Demographic and clinical characteristics of the nurses (*N* = 20).

ID	Age (Year)	Gender	Education Level	Clinical Experience with Patients of Hematology-Oncology Ward (y)	Total Clinical Experience (y)
1	26	Women	Bachelor’s	2.11	2.11
2	32	Women	Master’s	7.11	7.11
3	31	Women	Master’s	8.11	8.11
4	29	Women	Bachelor’s	6.60	6.60
5	29	Women	Bachelor’s	5.20	5.20
6	36	Women	Bachelor’s	11.00	14.30
7	36	Women	Bachelor’s	5.20	6.20
8	37	Women	Bachelor’s	9.00	14.00
9	30	Women	Associate’s	6.10	8.30
10	25	Women	Bachelor’s	3.00	3.00
11	26	Women	Bachelor’s	2.60	2.60
12	25	Women	Bachelor’s	2.80	2.80
13	26	Women	Bachelor’s	2.90	2.90
14	26	Women	Bachelor’s	2.11	2.11
15	25	Women	Bachelor’s	2.60	2.60
16	37	Women	Bachelor’s	14.11	14.11
17	27	Women	Bachelor’s	2.11	2.11
18	24	Women	Bachelor’s	2.00	2.00
19	24	Women	Bachelor’s	1.10	1.10
20	29	Women	Bachelor’s	5.40	5.40

**Table 2:** Categories, theme clusters, and themes of therapeutic communication experiences of nurses caring for patients with hematology.

Themes	Theme Clusters	Categories
. Understanding from a patient’s perspective based on values. Have an active response to the needs of the nurse	Empathize and take a proactive attitude from the patient’s perspective	Acquiring core competencies as nursing professionals
. Feel rewarded in a comfortable atmosphere. Build self-efficacy by helping patients	Improve satisfaction and self-efficacy through work	
. Feeling a reaction to separate emotions in an emotional situation	Staying centered so that nurses do not get carried away	
. Patient-centered treatment for positive effects	Promote active patient engagement	Improving patient-centered nursing performance
. Active expression of nursing needs. Creating a sympathetic atmosphere in tense situations	Relieve anxiety through rapport formation	
. Improving trust in nurses by providing professional practical skills and knowledge	Increase confidence in nurses’ work	
. Building a consensus on the idea of caring for family. Provide stability in the first hospital stay	Empathize and listen to the patient at their level	Forming a partnership treatment relationship
. Providing nursing care with respect for patients as people	Respect for the patient as a human being	
. Positive synergy effect in busy nursing situations. Patients affected by nurse behavior	Patient–nurse interaction	
. Self-development through the acquisition of professional knowledge and skills	Self-development	Obtain clinical performance skills to solve problems
. Feeling the need to provide educational opportunities according to communication and circumstances.. Expressing communication difficulties in different situations	Provide indirect experience opportunities for contextual scenarios	
. Adjusting the number of patients for the realization of full-time nursing. Difficulty in nursing work with increased severity	Efficient operation of nursing staff	Preparing efficient system improvement
. Sharing effective communication methods in different situations	Share experiences to improve nursing quality	
. Sharing information with other departments for patient care	Cooperate with other departments	

## Data Availability

Not applicable.
